# Influence of Impact Velocity on the Residual Stress, Tensile Strength, and Structural Properties of an Explosively Welded Composite Plate

**DOI:** 10.3390/ma13122686

**Published:** 2020-06-12

**Authors:** Aleksander Karolczuk, Krzysztof Kluger, Szymon Derda, Mariusz Prażmowski, Henryk Paul

**Affiliations:** 1 Department of Mechanics and Machine Design, Opole University of Technology, Mikołajczyka 5, 45-271 Opole, Poland; a.karolczuk@po.edu.pl (A.K.); szymon.derda@doktorant.po.edu.pl (S.D.); m.prazmowski@po.edu.pl (M.P.); 2 Institute of Metallurgy and Materials Science, Polish Academy of Sciences, Reymonta 25, 30-0 59 Kraków, Poland; h.paul@imim.pl

**Keywords:** explosive welding, residual stress, orbital hole-drilling strain-gauge method, prediction of tensile yield force, explosive cladding, Zr 700

## Abstract

This study aimed to analyze the effect of the impact velocity of a Zr 700 flyer plate explosively welded to a Ti Gr. 1/P265GH bimetallic composite on the residual stress formation, structural properties, and tensile strength. The residual stresses were determined by the orbital hole-drilling strain-gauge method in a surface layer of Zr 700 in as-received and as-welded conditions. The analysis of the tensile test results based on a force parallel to interfaces was used to propose a model for predicting the yield force of composite plates. Compressive residual stresses found in the initial state of the Zr 700 plate were transformed to tensile stresses on the surface layer of the welded Zr 700 plate. A higher impact velocity resulted in higher tensile stresses in the Zr 700 surface layer. To increase the resistance of the composite plate to stress-based corrosion cracking, a lower value of impact velocity is recommended in the welding process.

## 1. Introduction

Metallic composites belong to a group of materials in which the multilayer structure of different metallic alloys provides special functional properties [1]. Explosive cladding is one of the manufacturing processes used to produce multilayer metallic composites. It involves the energy of detonation to accelerate a flyer plate that, as a result, collides with a base plate [2,3]. High-velocity impact leads to the formation of a strong bond between colliding plates. Low-density materials, such as aluminum and magnesium alloys, can be explosively welded to steels and applied in the ship building and automotive industries [4,5]. A copper layer [6,7] within a composite multilayer material provides excellent electrical conductivity, and so-called reactive materials such as titanium [8,9], zirconium, and niobium exhibit corrosive resistance in an aggressive environment. This, in turn, predisposes them to wide usage in design applications in process equipment in particular [10]. A composition of tungsten foil and cooper layers is applicable for thermonuclear reactors [11] since it offers high resistance to heat loads and irritation.

Zirconium exhibits outstanding performance as a material for use in highly corrosive environments with a broad range of chemical media and temperatures [10,12,13]. This allows the material to be used in the chemical process industry for heat exchangers cooled with seawater and other pieces of process equipment as well as in nuclear-fuel-reprocessing plants. To reduce the financial cost of producing the process equipment, a zirconium alloy could be used as a relatively thin layer in cladded plates. According to Banker [10], replacing the solid structure with a zirconium wall of a thickness above 20 mm by a multilayer plate could have cost-effective benefits. Explosive welding is categorized within the solid-state process and is one of the ways to achieve a high-quality connection between dissimilar materials. 

The parameters of the explosive welding process determine the quality of the bond and the mechanical properties of the cladded plates. For higher explosive welding parameters, locally melted areas may be formed, which can involve brittle intermetallic compounds and shrinkage cracks [14,15]. Therefore, all parameters must be adequately selected to achieve optimal efficiency of the process. Such parameters include the physical properties of the welded materials, parameters of the explosive material, and the geometry of the welding setup. The explosively induced joint is inevitably associated with large material deformation, a significant temperature gradient, and rapid phase change in the surroundings of the impact zone. Therefore, residual stresses are locked-in stresses in the explosively welded plates [16,17]. The residual stress state may have a considerable effect on material performance. A compressive residual stress state is known to increase fatigue strength [18], and can be a cause of dimension instability during cutting or other manufacturing processes. In contrast, a tensile residual stress state tends to be undesirable as it accelerates crack growth and can induce stress-based corrosion cracking [19,20]. Nagano et al. [21] noted that a passive oxide film in pure zirconium and its alloys ruptured because of stress in corrosive environments depending on temperature and HNO_3_ concentration. Several other studies have reported on the stress-based corrosion cracking in zirconium and its alloys [22,23,24,25]. A high residual stress gradient can result in rapid delamination of welded plates within a few seconds after the collision of plates in the case of improper welding parameters. Even if the bond survives, the geometrical stability of the welded plate during cutting can be lost. In the case of the application of multilayer plates in processing equipment, the possible failure of the corrosive resistance layer is unacceptable as it would lead to undetected corrosion because of the release of corrosive compounds throughout the backing material. To reduce the failure probability of the corrosive resistance layer, the residual stresses in the near-surface layer should be as low as possible, with a preferably compressive character. However, the problem of the influence of explosive welding parameters on residual stress states in the zirconium layer of composite plates was not profoundly analyzed. 

The present study is aimed at analyzing the influence of impact velocity on the tensile strength, the structural properties of composite plates, and the generation of residual stress in the flyer plate (a plate with explosive charge) made of Zr 700 alloy. Two impact velocities were achieved by altering the stand-off distance by using a fixed quantity of the explosive charge. 

Presently, limited research has been devoted to the determination of residual stress in composite structures obtained through the explosive welding process. Due to the wide range of analyzed composite structures and the use of different methods to determine residual stresses, unambiguous conclusions on the state of residual stress in explosively welded multilayer structures cannot be deduced. In most cases, tensile residual stresses were detected in the surface layer of the flyer plate [16,26,27,28,29,30,31]. Limited cases with compressive residual stresses were found [17,32,33]. The hole-drilling strain-gauge method seems to be the most relevant, as it is included in the ASTM standard [34]. In addition, both the limitation and accuracy of this method are well established and are available in the literature.

In this study, the residual stresses were determined by employing the hole-drilling strain-gauge method recommended by the ASTM standard [34]. The stresses were determined in the Zr 700 layer for two plates welded under different impact velocities. Additionally, the residual stresses in the Zr 700 plate before welding were determined and used as the reference value. 

The characteristic features of explosively welded materials include a wavy character of the interface between joint metals and locally melted areas. These features of the interface were described by measuring the sizes of the melted areas as well as the height and length of the interface wave. The data were obtained through the microhardness measurement across welded plates. In addition, tensile tests were conducted with force applied in the parallel direction to the interface. Moreover, a model for predicting the yield force of the composite plate is proposed.

## 2. Experiment

### 2.1. Materials in As-Delivered Condition

A composite structure consisting of three layers made of P265GH pressure vessel steel, Ti Gr. 1, and zirconium Zr 700 was manufactured in the explosive welding process. Table 1 and Table 2 summarize the chemical composition and mechanical properties of the materials examined in this study, respectively. The tensile tests were conducted to estimate the presented mechanical properties.

**Figure 1:**
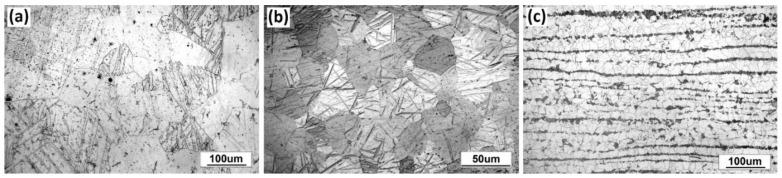
Microstructure of materials in as-delivered conditions: (**a**) Zr 700 alloy, (**b**) Ti Gr. 1 alloy, and (**c**) P265GH steel.

The microstructure of the materials under as-delivered conditions is illustrated in Figure 1. 

The microstructure of Zr 700 plate in the section perpendicular to the rolling direction is presented in Figure 1a. The material was characterized by the structure of the α-phased grains sized between 70 and 170 µm. The microstructure of the material used in the interlayer, i.e., Ti Gr. 1 alloy, consisted of α-phased equiaxed grains sized between 20 and 40 µm (Figure 1b). Figure 1c presents the P265GH carbon steel microstructure characterized by an equiaxed structure of mid-sized grains of 4–11 and 10–20 µm for pearlite and ferrite, respectively. As shown, a band structure composed of fine grains of pearlite was observed; this is a typical phenomenon for materials after the hot-forming process.

### 2.2. Explosive Welding Process

The explosive welding process was conducted by High Energy Technology Works “Explomet” (Opole, Poland). Consequently, two multilayer plates were produced with different welding parameters. In both cases, the flyer plate made of Zr 700 with a thickness of 10 mm was cladded to the preliminary welded bimetallic plate composed of a 2-mm Ti Gr. 1 layer and a 14-mm P265GH steel layer. The plates with dimensions of 300 mm × 500 mm were welded in parallel by applying an explosive charge of ammonites, with NH_4_NO_3_ (High Energy Technology Works “Explomet”, Opole, Poland) as the main component. The detailed composition of the explosive charge was not provided by the supplier of the composite plates. The applied explosive charge resulted in a detonation velocity of $v_{D}=2500$ m/s, which was measured using a fiber optic system [36]. The welding processes for the studied plates have different values of stand-off distance δ (which is the initial distance between the flyer plate, Zr 700, and the basic Ti Gr. 1–P265GH bimetal). For the first plate, labeled as B3, the stand-off distance was δ = 10 mm, whereas it was δ = 15 mm for the second plate (B4). The application of different δ values resulted in different impact velocities $v_{P}$ estimated using the Deribas formula [37,38]. The summarized welding parameters are presented in Table 3.

**Figure 2:**
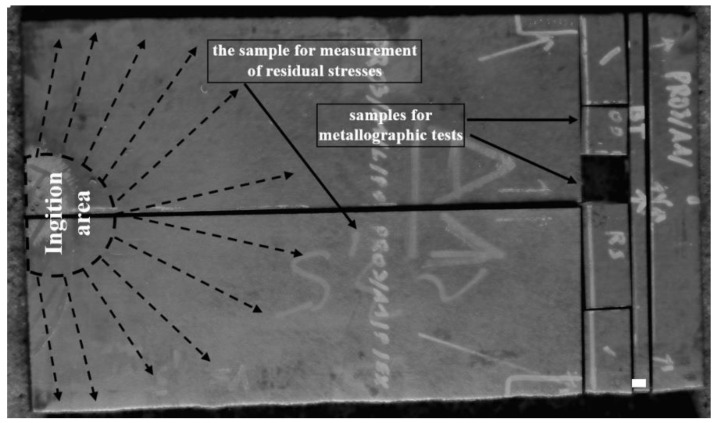
Explosively welded plate with the marked ignition area and samples for residual stress estimation and microstructural analysis.

Both the welded plates underwent the flattening process and ultrasonic examination [39], revealing no discontinuities except near the ignition and narrow area at the plate edges (approximately 20 mm from the edge). Figure 2 shows the locations of the ignition point and samples used for residual stress measurement and microstructural analysis. 

### 2.3. Residual Stress Estimation

Residual stress was identified using the incremental hole-drilling strain-gage method recommended in the ASTM E837-13a standard [34], and the experiments were conducted on the surface of the flyer Zr 700 plate. Additionally, for referential analysis, residual stresses in the Zr 700 plate in the as-delivered condition were estimated. 

For measuring the relieved strains during incremental hole drilling, a Vishay RS-200 device (Vishay Precision Group, Malvern, PA, USA) with a pneumatic turbine was used in orbital drilling mode. This method appears to be beneficial in several ways, one of which is the considerable improvement of drilling conditions [40]. In addition, a Vishay milling cutter (Vishay Precision Group, Malvern, PA, USA) was used for the drilling and producing holes with the final diameter of approximately 1.9 mm. The holes were drilled in incremental steps of 0.05 mm to a total depth of 1 mm. This study employed a three-element type-A [34] strain rosette gauge of FRS-2 (TML Lab. Company, Tokyo, Japan), paired with a multi-channel signal-acquisition device (P3 Strain Indicator and Recorder, Vishay Precision Group, Malvern, PA, USA) (with resolution of strain measurement equal to 10^−6^). Four measurement points were located in the middle part of plate B3 and two on plate B4. The distance between the points varied from 30 to 70 mm. Additional residual stress estimation for the Zr 700 plate under the as-delivered condition was based on two measurement points. 

### 2.4. Structural Properties

**Figure 3:**
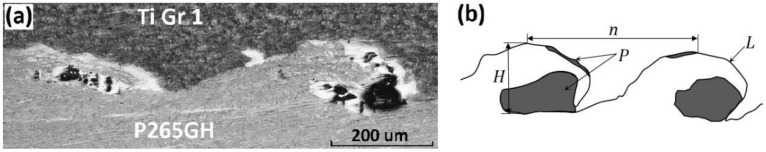
(**a**) Interfacial wave of plate B4, and (**b**) structural properties of the wavy interface: length of the welded line (L, wave height (H), wavelength (n), and melted area (P).

The process of explosive welding is characterized by the formation of an interface between the periodic deformation and the interfacial wave [3] (Figure 3a). 

The geometrical parameters of the wave depend on the process parameters and physical properties of the welded materials [7,41,42,43]. These parameters could be correlated with mechanical properties of the composite structure [7,44,45]. For some welding systems, a melted area can be formed in the vortex of the collision zone. In this study, the following geometrical quantities were measured: wave height H, wavelength n, and melted area P along the length of welded line L. Figure 3b illustrates the measurement of the geometrical parameters of the samples cut out from both welded plates by using the digital optical method according. The total length of welded line L varies between 15 and 18 mm, including 8–12 points for wavelength and wave height determination for the Zr 700–Ti Gr. 1 interface and 20–37 points for the Ti Gr. 1–P265GH interface. Analyzed data were collected to calculate the mean and standard deviation for each parameter. Following a previous study [7], the authors calculated a parameter that describes the averaged amount of melted area: equivalent melted thickness, EMT=P/L. 

In addition to the geometrical properties, microhardness distribution was included as a structural property. The microhardness distribution provides information on induced material hardening due to severe plastic deformation that occurred during the impact of two plates. The distribution of Vickers microhardness (HV) under the 50-G load was measured along three lines perpendicular to interfaces with the distance of 0.06 mm between points in the vicinity of the welded zone. The final results were obtained as the mean values of the three measurements (three lines) supplemented with error bars representing standard deviations. The HV hardness of the as-delivered plates was also measured for comparison.

### 2.5. Mechanical Test

**Figure 4:**
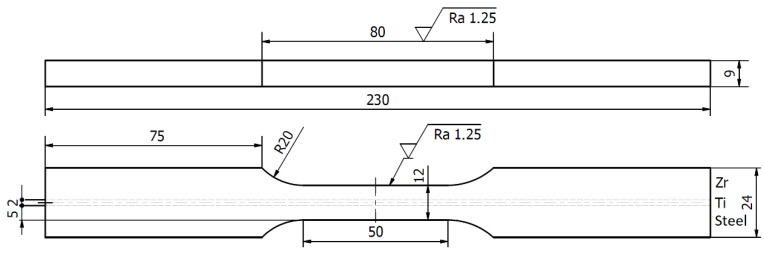
Geometry of specimen for the tensile test (units in mm).

The tensile test was designed to verify the strength of the cross-section perpendicular to the interfaces. Figure 4 presents the geometry of the specimen used for the tensile test. 

The test facilitated the determination of force $F_{p02}$ for the 0.2% offset of plastic strain, ultimate force $F_{m}$, and elongation to rupture A. The obtained values for plate B4 and its composite plates can be compared. These results can be verified using theoretical values calculated based on the mechanical properties of materials in the as-delivered condition. 

## 3. Calculation, Results, and Discussion

### 3.1. Residual Stresses

**Figure 5:**
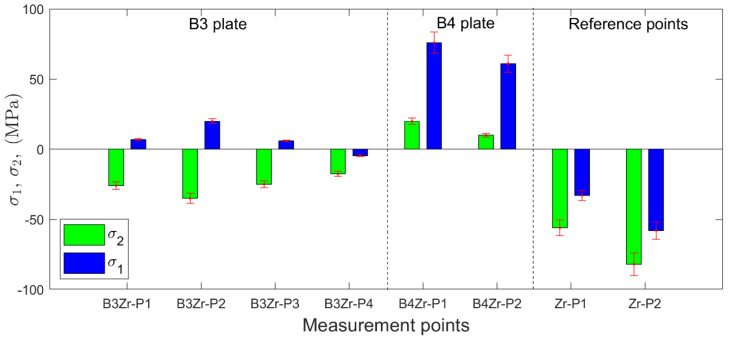
Residual stresses identified in the Zr 700 layer of B3 (points P1–P4), B4 (points P1 and P2), and Zr 700 plate before welding (reference points P1 and P2).

The relieved strain components recorded during the incremental drilling were evaluated and recalculated with respect to residual principal stresses by using the Eval 7 software (Sint Technology). This software is fully compliant with the international ASTM E837-13a standard [34] for residual stress measurement using the hole-drilling strain-gage method. The software also allows evaluation of the uncertainty of stress calculation [46]. The following accuracies of material and drilling parameters were found: Young’s modulus = ±3 GPa, Poisson ratio = ±0.01, strain = ±0.6 μm/m, strain gauge factor = ±1%, hole diameter = ±0.04 mm, and hole depth = ±0.025 mm. A uniform stress field was assumed throughout the calculations. The obtained maximum ($\sigma_{1}$) and minimum ($\sigma_{2}$) principal stresses may be considered as average values along the 1-mm hole depth, i.e., near the surface layer of Zr 700 plate. The results of the residual stresses determined in the Zr 700 layers of composite plates B3 and B4 as well as the reference stresses determined for the Zr 700 plate before welding are presented in Figure 5. 

The principal residual stresses determined for the as-delivered condition in the Zr 700 plate represent the state of the residual stress before welding (reference points in Figure 5). This biaxial compressive stress state with component values between −82 ± 8 MPa and −33 ± 4 MPa was transformed into the biaxial tensile stress state (10 ± 1 MPa and 76 ± 8 MPa) for composite plate B4. In contrast, composite plate B3, produced with a lower impact velocity of $V_{p}=425\;m/s$ as compared to plate B4 ($V_{p}=468\;m/s$), indicated lower values of residual stresses, ranging between −35 ± 4 MPa and 20 ± 2 MPa. The performed manufacturing processes had a considerable effect on the residual stress state. The initial compressive stress state, which was beneficial in terms of stress-based corrosion cracking, became more tensile with the increasing magnitude of impact velocity.

### 3.2. Structural Properties

**Figure 6:**
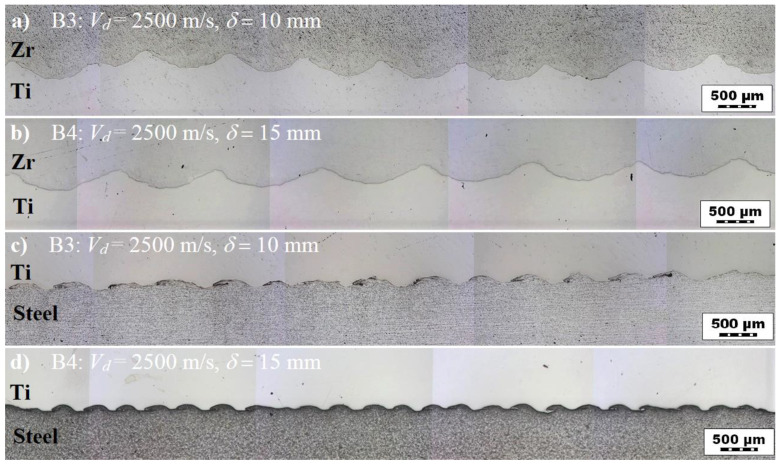
Morphology of interfacial waves for composite plates B3 and B4. Zr–Ti interface for (**a**) plate B3 and (**b**) plate B4. Ti–steel interface for (**c**) plate B3 and (**d**) plate B4.

**Figure 7:**
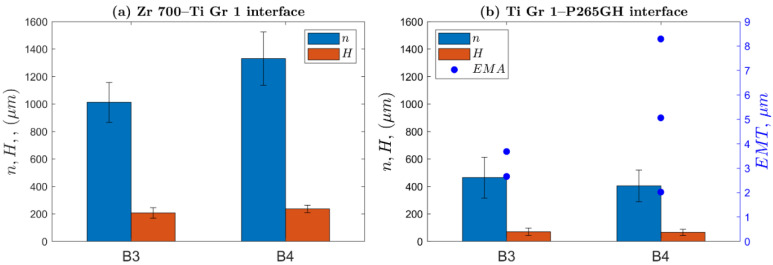
Wavelengths n and wave heights H for (**a**) the Zr 700–Ti Gr. 1 interface and (**b**) the Ti Gr. 1–P265GH interface with an equivalent thickness of melted area (EMT).

Figure 6 presents the morphologies of the interfacial waves for composite plates B3 and B4. As shown, the interfacial waves of the Zr 700–Ti Gr. 1 joint in both the plates did not exhibit melted areas (EMT=0 μm) but showed higher values for the wavelength and wave height than those observed for the Ti Gr. 1–P265GH joint. The measured parameters are presented in Figure 7. Compared to plate B3, the higher impact velocity (by 10%) for plate B4 resulted in higher wavelength (by 31%) and wave height (by 14%) at the Zr 700–Ti Gr. 1 interface. At the Ti Gr. 1–P265GH interface, the wave parameters were nearly equal (within the standard deviation band). The EMT values were low [45], at approximately 3 and 5 μm for plates B3 and B4, respectively. 

**Figure 8:**
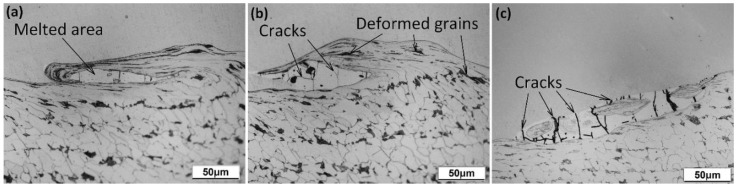
Microstructure with grain deformation and microcracks observed in melted areas of (**a**) plate B3 and (**b**,**c**) plate B4 in different locations.

**Figure 9:**
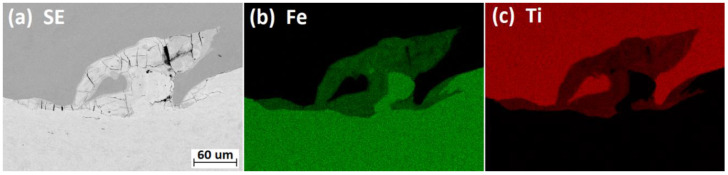
(**a**) SEM (Scanning Electron Microscope) and (**b**,**c**) EDX (Energy Dispersive X-Ray Analysis) maps showing the distribution of Fe and Ti at the Ti Gr. 1–P265GH interface for plate B4.

The melted areas at the Ti Gr. 1–P265GH interface were localized mainly in the vortex formed as a result of fluid–structure interaction (Figure 8.). The melted areas comprise a new phase with mixed chemical composition of Ti and Fe (Figure 9). In both plates B3 and B4, multiple microcracks (Figure 8 and Figure 9a) were detected in the melted areas; these were probably formed owing to severe residual stresses generated during the rapid cooling rate (shrinkage cracks [14]). Furthermore, grain deformation was observed to intensify closer to the weld line. 

**Figure 10:**
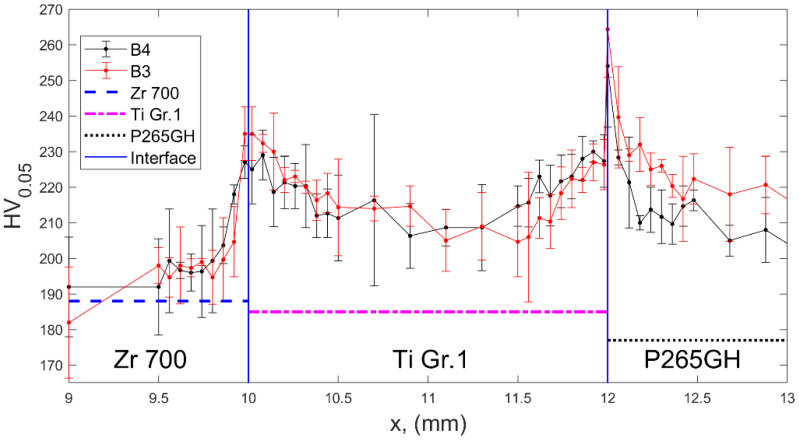
Vickers microhardness distribution across the composite plates, with horizontal lines representing hardness of materials in the as-delivered conditions.

The microhardness distributions presented in Figure 10 exhibited an increase in the plate hardness in close vicinity of interfaces. In both plates B3 and B4, the distributions of microhardness were similar within the standard-deviation error bands. The highest value of microhardness of 250 HV_0.05_ was detected in the Ti Gr 1–P265GH interface; it exceeded the hardness of steel by approximately 40%. 

### 3.3. Mechanical Test

**Figure 11:**
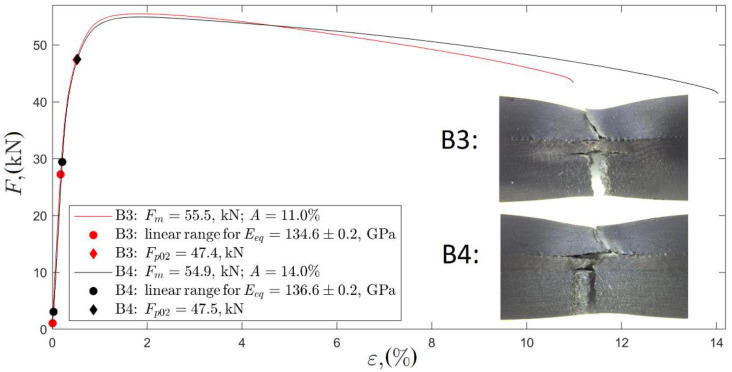
Strain–force curves of tensile test conducted on composite plates B3 and B4.

Figure 11 presents the results of tensile tests represented as strain–force curves. The curves for the specimens made of composite plates B3 and B4 differ insignificantly. 

The estimated curve parameters of yield force $F_{p02}$, ultimate force $F_{m}$, elongation A, and equivalent Young’s modulus $E_{eq}$ were calculated, as shown in Table 4. The equivalent Young’s modulus was determined for the linear range of the curve as force F divided by total cross-section area A and strain ε, $E_{eq}=F/\left(A\varepsilon \right)$. Additionally, the fractures of both plates are presented in Figure 11.

**Figure 12:**
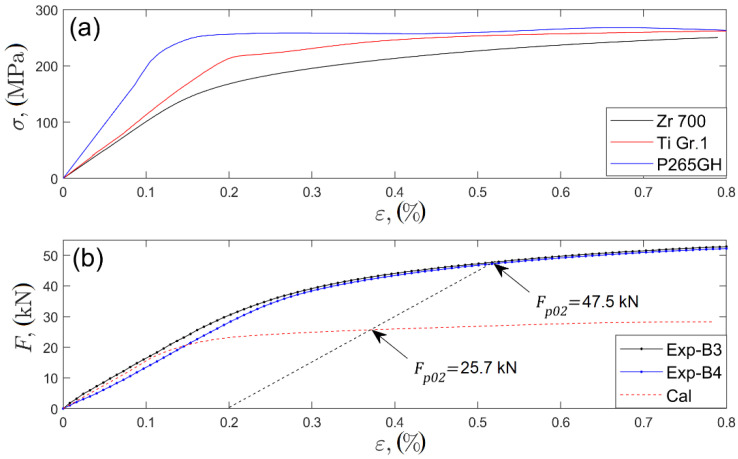
(**a**) Recorded strain stress curves for materials in as-delivered condition. (**b**) Experimental force–strain curves for plates B3 and B4 (Exp-B3 and Exp-B4) and the calculated curve.

The strain–force curve was estimated theoretically to quantify the obtained results with respect to the possible increase of strength properties [47] in relation to material properties in the as-delivered condition. The following assumptions were made in the calculation model: (i) the cross-section of the specimen (Figure 4) comprised uniform strain distribution and (ii) all the layers comprised the uniaxial stress state. This model applies constitutive empirical relations of $\sigma^{Zr}-\varepsilon^{Zr}$, $\sigma^{Ti}-\varepsilon^{Ti}$, and $\sigma^{St}-\varepsilon^{St}$ of materials in the as-delivered condition (tensile tests). The recorded strain signal, ε, for the composite plate was used to estimate the stress in each layer through the following empirical constitutive relations: $\sigma^{Zr}\left(\varepsilon =\varepsilon^{Zr}\right)$, $\sigma^{Ti}\left(\varepsilon =\varepsilon^{Ti}\right)$, and $\sigma^{St}\left(\varepsilon =\varepsilon^{St}\right)$. The stresses $\sigma^{Zr},\sigma^{Ti},$ and $\sigma^{St}$ estimated through the force balance equation in each layer were used to determine force value F:

(1) $$F=\overset{3}{\underset{i=1}{\sum }}\sigma^{i}A^{i}=\sigma^{Zr}A^{Zr}+\sigma^{Ti}A^{Ti}+\sigma^{St}A^{St},$$

where $A_{Zr},A_{Ti},$ and $A_{St}$ are the initial cross-sectional areas of the Zr 700, Ti Gr 1, and P265GH layers, respectively. The empirical strain–stress curves for the material in the as-welded condition are presented in Figure 12a.

The model implemented the initial cross-sectional areas, and thus its applicability was limited to a small strain regime (assumed to be less than 1%). The slope of the calculated curve in the linear range (up to ~20 kN; Figure 12b) is in accordance with the slope of the experimental curves for both plates B3 and B4. Computed yield force $F_{p02}=25.7\;\mathrm{kN}$ was considerably lower than its empirical value of $F_{p02}=47.5\;\mathrm{kN}$. The increase in the yield force by approximately 85% was due to material hardening in the vicinity of the interfaces. The research presented in [48,49,50] showed that the yield strength proportionally increased with the hardness of different types of steels and zirconium alloys [51]. The yield force $F_{p02}$ of explosively welded plates is proposed to be predicted according to the proportional increase in yield stress $R_{p02}$ as the function of hardness rate:

(2) $$F_{p02}=\overset{3}{\underset{i=1}{\sum }}c^{i}r_{HV}^{i}R_{p02}^{i}A^{i}=c^{Zr}r_{HV}^{Zr}R_{p02}^{Zr}A^{Zr}+c^{Ti}r_{HV}^{Ti}R_{p02}^{Ti}A^{Ti}+c^{St}r_{HV}^{St}R_{p02}^{St}A^{St},$$

where $c^{i}\;$ represents the proportionality factors for each layer (i=[Zr, Ti, St]), and $r_{HV}^{i}=HV_{h}^{i}/HV_{0}^{i}$ represents the hardness rates (hardness of the hardened material $HV_{h}^{i}$ divided by the hardness of the material in the initial state $HV_{0}^{i}$). The hardness of $HV_{h}^{i}$ was calculated as the average hardness of each layer (5 mm + 2 mm + 5 mm). The calculated hardness rates were as follows: $r_{HV}^{Zr}=0.97$, $r_{HV}^{Ti}=1.16$, and $r_{HV}^{St}=1.25$. The proportionality factors were assumed to be equal, i.e., $c^{Zr}=c^{Ti}=c^{St}=c$. Proportionality factor c=1.58 was identified by fitting the experimental and calculated (Equation (2)) yield forces.

The process of explosive welding includes problems of phase transformation, shock impact, plastic deformation, and fluid–structure interaction. A thorough analysis of the process requires sophisticated multiphysics numerical modeling [52,53,54,55], which is nowadays narrowed to the prediction of jet formation, morphology of the interfacial wave, and weldability. Residual stress formation has not yet been numerically simulated. However, based on the knowledge of the explosive welding process, a general scheme of residual stress generation can be proposed. During the shock of impact of the Zr 700 plate with the Ti Gr. 1 layer, a large hydrostatic compressive stress due to inertial forces could be formed in the contact region. The increase of local temperature reduces yield stress of both materials in contact, allowing the occurrence of plastic deformation, which is sometimes manifested by the adiabatic shear bands [8,56,57]. More intensive, larger, and plastically deformed regions appear in a material with lower resistance to plastic yield. In most cases, the material with a lower yield strength and higher elongation is selected as the flyer. In this case, larger and more intensive plastic deformations occur in the flyer region in the vicinity of impact, and more elastic potential energy is stored in the base plate. When the pressure of explosive gas is released, the elastically deformed base plate below the interface expands and compresses the plastically deformed layer of the flyer plate. The induced compressive residual stresses in the vicinity of the interface must be balanced by the tensile residual stresses formed in the outer layer of the flyer plate (as a result of the spring-back effect [3]). The locally melted areas solidify and form a new phase; these areas are the sources of thermal residual stresses, which deviate from the general field of stresses described. The higher the differences in Young’s modulus and thermal expansion coefficients, the higher the residual stresses. Stress relief with respect to heat treatment is possible only for materials with close values of thermal expansion coefficients. 

In this study, the difference in the impact velocities by approximately 10% resulted in insignificant differences in microhardness distribution and tensile yield force of specimens with reduced thickness (5 mm + 2 mm + 5 mm). In addition, a notable increase of the wavelength by approximately 31% was observed. In contrast to most structural properties, the residual stresses in a 1-mm-thick Zr 700 surface layer exhibited profound sensitivity to the applied impact velocities. The initial compressive residual stress state was significantly transformed towards the tensile type, reaching 76 ± 8 MPa for higher impact velocities. 

## 4. Conclusions

The main conclusions of the study are summarized as follows:

- The compressive residual stress, which was initially present in the Zr 700 flyer plate, decreased in the explosive welding process, resulting in a tensile type with an increase in impact velocity.
- To protect the composite plate from stress-based corrosion cracking, a lower value of the impact velocity is recommended.
- The experimental yield force of composite specimens is around 85% higher than the yield force of combined properties of materials in the as-delivered condition.
- The experimentally estimated residual stresses could be used to verify the numerical method applied in modeling of the explosive welding process.
- In addition, a simple model based on microhardness measurement for yield force prediction of the composite plate was proposed. However, the model needs further verification.

## Figures and Tables

**Table 1:** Chemical composition of materials in as-delivered conditions [35].

Materials	Chemical Composition (wt %)											
P265GH	Mn 0.959	Si 0.260	C 0.147	Al 0.051	Ni 0.030	Cr 0.022	P 0.011	Nb 0.008	S 0.006	Mo 0.005	N 0.004	Fe Balance
Zr 700	O 0.067		Fe 0.060		C 0.004		N <0.002		H <0.0003		Zr + Hf Balance	
Ti Gr. 1	O 0.070		F 0.020		C 0.020		N <0.010		H 0.010		Ti Balance	

**Table 2:** Basic mechanical properties of applied materials.

Material	E, (GPa)	ν, (-)	$R_{p02},\;(\mathrm{MPa})$	$R_{m},\;(\mathrm{MPa})$	A, (%)
Zr 700	101	0.38	216	269	35
Ti Gr. 1	109	0.37	251	325	46
P265GH	193	0.29	268	391	41

E is the elastic modulus, ν is Poisson’s ratio, A is the elongation, $R_{p02}$ is the yield strength, and $R_{m}$ is the tensile strength.

**Table 3:** Explosive welding parameters.

Plate	Flyer	Thickness, (mm)	Detonation Velocity, $v_{D,}\;\left(\mathrm{mm}\right)$	Stand-off Distance, δ, (mm)	Impact Velocity $v_{p},\;\left(m/s\right)$
B3	Zr 700	10	2500	10	425
B4	Zr 700	10	2500	15	468

**Table 4:** Results of tensile tests on composite plates B3 and B4.

Plate	$F_{p02},\;(\mathrm{kN})$	$F_{m},\;(\mathrm{kN})$	A, (%)	$E_{eq},\;(\mathrm{GPa})$
B3	47.4	55.5	11	134.6
B4	47.5	54.9	14	136.6
